# Inherited breast cancer predisposition in Asians: multigene panel testing outcomes from Singapore

**DOI:** 10.1038/npjgenmed.2015.3

**Published:** 2016-01-13

**Authors:** Edward S Y Wong, Sandhya Shekar, Marie Met-Domestici, Claire Chan, Melody Sze, Yoon Sim Yap, Steven G Rozen, Min-Han Tan, Peter Ang, Joanne Ngeow, Ann S G Lee

**Affiliations:** 1 Division of Medical Sciences, Humphrey Oei Institute of Cancer Research, National Cancer Centre, Singapore, Singapore; 2 Division of Medical Oncology, National Cancer Centre Singapore, Singapore, Singapore; 3 Oncology Academic Clinical Program, Duke-NUS Graduate Medical School, Singapore, Singapore; 4 Department of Medicine, Yong Loo Lin School of Medicine, National University of Singapore, Singapore, Singapore; 5 Centre for Computational Biology, Duke-NUS Graduate Medical School, Singapore, Singapore; 6 Division of Biodevices and Diagnostics, Institute for Bioengineering and Nanotechnology, Singapore, Singapore; 7 OncoCare Cancer Centre, Mount Elizabeth Novena Specialist Centre, Singapore, Singapore; 8 Department of Physiology, Yong Loo Lin School of Medicine, National University of Singapore, Singapore, Singapore; 9 Office of Clinical & Academic Faculty Affairs, Duke-NUS Graduate Medical School, Singapore, Singapore

## Abstract

Genetic testing for germline mutations in breast cancer predisposition genes can potentially identify individuals at a high risk of developing breast and/or ovarian cancer. There is a paucity of such mutational information for Asians. Panel testing of 25 cancer susceptibility genes and *BRCA1/2* deletion/duplication analysis was performed for 220 Asian breast cancer patients or their family members referred for genetics risk assessment. All 220 participants had at least one high-risk feature: having a family history of breast and/or ovarian cancer in first- and/or second-degree relatives; having breast and ovarian cancer in the same individual or bilateral breast cancer; having early-onset breast cancer or ovarian cancer (⩽40 years of age). We identified 67 pathogenic variants in 66 (30.0%) patients. Of these, 19 (28.3%) occurred in *BRCA1*, 16 (23.9%) in *BRCA2*, 7 (10.4%) in *PALB2*, 6 (9.0%) in *TP53*, 2 (3.0%) in *PTEN*, 2 (3.0%) in *CDH1* and 15 (22.4%) in other predisposition genes. Notably, 47.8% of pathogenic variants were in non-*BRCA1/2* genes. Of the 66 patients with pathogenic mutations, 63.6% (42/66) were under the age of 40 years. Family history of breast and/or ovarian cancer is enriched in patients with *BRCA1/2* pathogenic variants but less predictive for non-*BRCA1/2* related pathogenic variations. We detected a median of three variants of unknown significance (VUS) per gene (range 0–21). Custom gene panel testing is feasible and useful for the detection of pathogenic mutations and should be done in the setting of a formal clinical cancer genetics service given the rate of VUS.

## INTRODUCTION

In this era of precision medicine, gene-directed risk stratification and management is a common aspiration for modern clinical practice.^1^ This is reflected in the U.S. Department of Health and Human Services’ genomic objectives of Healthy People 2020 emphasising the importance of obtaining a family and genetic history as a potential and powerful guide for clinical and public health initiatives. The first genomic recommendation is that women with a family history of breast or ovarian cancer should receive genetic counselling. These genomic recommendations are based on the premise that gene-enabled management could improve health outcomes of affected individuals and allow family members to make proactive choices with their health. Indeed, at the recently launched *BRCA* Challenge at UNESCO, global expert faculty met to discuss ways to expedite this process through data sharing and to address the urgent need for data from diverse populations such as ours (http://www.unesco.org/new/en/media-services/single-view/news/breast_cancer_brca_challenge_officially_launched/).

Breast cancer susceptibility is associated with germline mutations in several genes such as *BRCA1*, *BRCA2*, *PTEN*, *TP53*, *PALB2, CDH1* and *STK11,* and genes of moderate penetrance like *ATM* and *CHEK2*.^2^ Next-generation sequencing (NGS) technology has enabled panel based genetic testing to the clinic, providing cost savings and the ability to test many genes simultaneously.^3^ However, the disadvantage of panel testing is the increased probability of encountering a germline VUS. This is particularly problematic in minority populations where there is less data available and/or in regions where the uptake of testing has been traditionally slow, such as in Asia. We present here the largest study undertaken to assess the use of NGS panel testing for breast cancer susceptibility genes in an Asian multi-racial cohort of patients referred for genetic risk assessment in Singapore.

## RESULTS

### Study population

Patients suspected of hereditary breast cancer in this study were referred from Singapore and the region for genetic risk assessment at the National Cancer Centre Singapore. Of the patients with established ethnicity, 181 (82.3%) were Chinese, 17 (7.7%) Malay, and 6 (2.7%) of South Indian descent (Table 1). The remaining 16 (7.3%) were of Burmese, Eurasian, Japanese, Filipino, Vietnamese and other races, respectively. Age at diagnosis of patients with breast and/or ovarian cancer ranged from 19 to 72 years, with an average age of 39 years. Of the 120 patients with available family history information, 104 (86.7%) had at least one first- or second-degree relative with breast cancer, and 16 (13.3%) had a relative with ovarian cancer.

### Germline mutations

**Figure 1:**
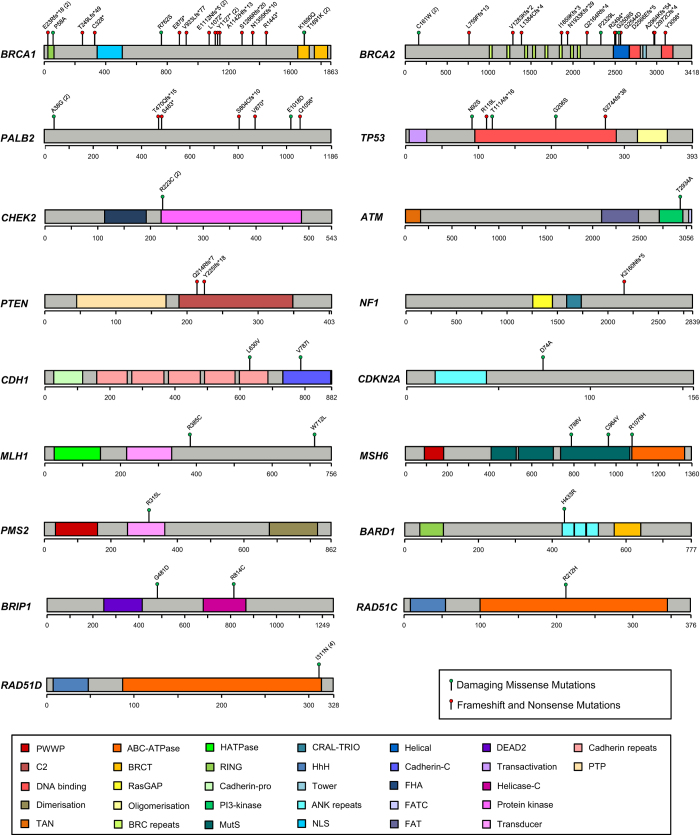
Pathogenic variants detected in 17 genes.

**Figure 2:**
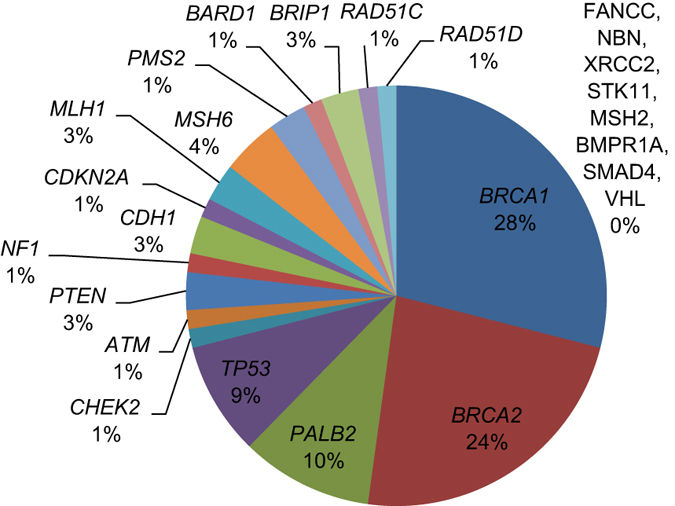
Pie-chart showing the percentage of mutations across the 25 genes.

All coding exons and consensus splice sites of 25 known cancer predisposition genes were screened for mutations in the 220 patients. Overall, 67 pathogenic mutations were identified in 66 patients (30.0% (66/220); Table 2). Eight mutations were detected in more than 1 patient, and 10 patients were carriers for more than one mutation (Table 2). Of these, 19 (28.4%) occurred in *BRCA1*, 16 (23.9%) in *BRCA2*, 7 (10.4%) in *PALB2*, 6 (9.0%) in *TP53*, 2 (3.0%) in *PTEN,* 2 (3.0%) in *CDH1* and 15 (22.4%) in other predisposition genes (Table 2; Figures 1 and 2). Deleterious *BRCA1* mutations were detected in 10.5% (23/220) of patients, including 15 truncating (frameshift, nonsense and splice, large deletion/duplication) mutations and 3 known deleterious missense mutations and 1 novel missense mutation. The 16 deleterious *BRCA2* mutations (7.7% (17/220)) included 12 truncating mutations, and 4 predicted deleterious missense mutations. Likely deleterious mutations in non-*BRCA1/2* predisposition genes were identified in 14.5% of all tested patients (32/220) in the following genes *ATM*, *BARD1*, *BRIP1*, *CDH1*, *CDKN2A*, *CHEK2*, *MLH1*, *MSH6*, *NF1*, *PALB2*, *PMS2*, *PTEN*, *RAD51C*, *RAD51D* and *TP53*. A total of 28 novel potentially pathogenic variants were detected in *BRCA1*, *BRCA2*, *PALB2*, *TP53*, *PTEN*, *NF1*, *CDH1*, *MSH6* and *PMS2* (Table 2) by our group in this study and previous studies.^4–6^

The mean Manchester score among cases with deleterious mutations was 19.4 (range 1–75) which was higher as compared to cases with no deleterious mutations (mean 9.7; range 1–71). Manchester scores were available for 56 of 66 individuals with deleterious mutations, and 124 of 154 individuals with no mutations.

### Family history

We also evaluated whether patients with mutations in the 25 predisposition genes were associated with a greater family history of breast and/or ovarian cancers than non-mutated patient cases (Table 2). Patients with *BRCA1* mutations were enriched for a family history of breast (5/23 (21.7%)) and ovarian cancers (2/23 (8.7%)), whereas patient cases with *BRCA2* mutations were enriched for a family history of breast (7/17 (41.2%)) but none of the family members had ovarian cancers. (Table 2). This is reflected in the differences in Manchester and Boadicea scores seen between the two groups of patients (Table 3). However, patient cases with mutations in the non-*BRCA1/2* genes were not significantly associated with an enriched family history for either breast or ovarian cancer (Table 2). In particular, only 8 (24.2% (8/33)) non-*BRCA1/2* gene mutation carriers had a family history of breast or ovarian cancer.

### Variants of unknown significance

**Figure 3:**
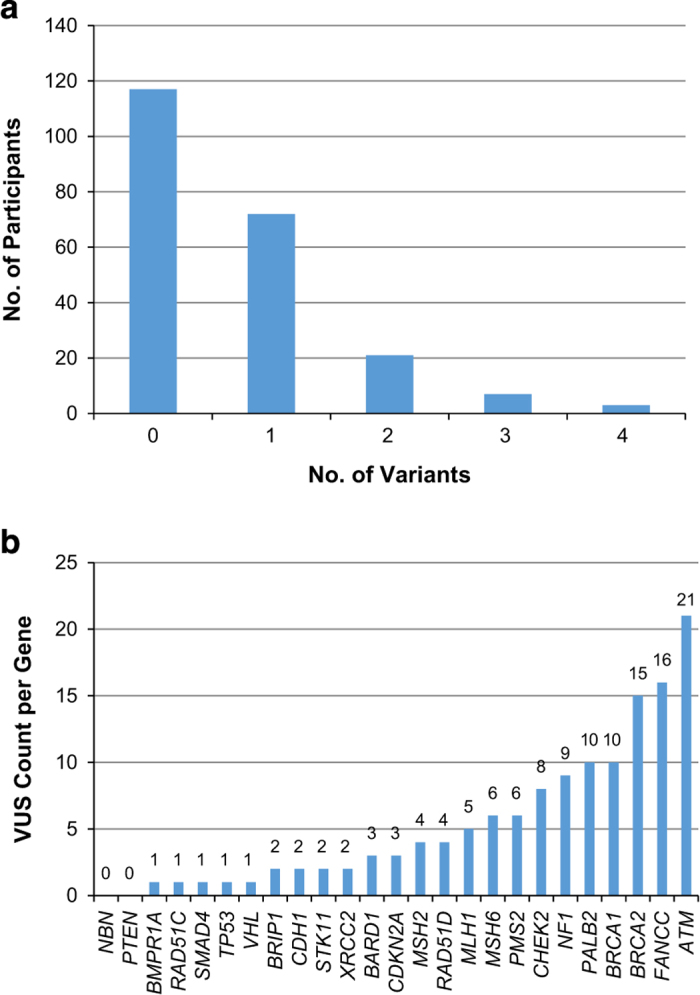
Frequency of variants of uncertain significance (VUS). (**a**) per participant, across 25 sequenced genes; and (**b**) per gene, across 220 participants.

A total of 94 VUS were identified in 23 genes in 96 of 220 participants. Per participant, the average number of VUS across all genes was 0.67 (s.d., 0.9) (Figure 3a). Of the 220 participants, 103 (46.8%) had at least one VUS among the 25 genes sequenced. Per gene, the median number of VUS detected across all 220 participants was 3, ranging from zero (*PTEN* and *NBN*) to 21 (*ATM*; Figure 3b). Among the 7 high-risk genes, 10 VUS were found in *BRCA1,* 15 in *BRCA2*, 10 in *PALB2,* 2 in *CDH1*, 2 in *STK11*, 1 in *TP53* and none in *PTEN*. In the remaining 18 genes, a median of 3.5 VUS per gene (range 0–21) were detected. All VUS were missense mutations and within exonic regions. Of the 94 VUS, 41 (43.6%) were novel, not previously reported in the databases or dbSNP. No statistically significant difference was detected in VUS frequency between ethnicities.

## DISCUSSION

We present here a comprehensive mutation analysis of Asian patients suspected of having hereditary breast cancer. To our knowledge, this is the largest Asian series to date for the NGS screening of germline mutations using a panel of known breast cancer predisposition genes. We found 67 germline deleterious mutations in 17 of 25 predisposition genes tested. *BRCA1* and *BRCA2* mutations were found in 17.7% (39/220) of patients, consistent with other studies using panel testing, whereas mutations in 15 other genes were found in 32 (14.5%) patients. The frequency of these mutations, especially in *PALB2*, which has recently been associated with a high lifetime risk of breast cancer, was similar to the frequency in high- and moderate-risk breast cancer families.^7^ This is a significant higher yield of potentially actionable results, compared with the 5 to 10% probability threshold endorsed by guidelines for testing for HBOC and Lynch syndrome testing.

In Asia and many parts of the world, while there is a growing appreciation for the testing of patients identified as being at high risk of hereditary cancer, it is still not as yet ‘mainstream’ practice, as such patients are often referred after the development of multiple cancers in a patient. This may account for the relatively high number of *TP53* (9.0%) and *PTEN* (3.0%) germline mutations seen in our cohort. Notably, only 63.6% (42/66) of patients with pathogenic variants were under the age of 40 years at the age of first cancer diagnosis, suggesting that age alone as a cut-off may miss significant numbers of patients (Table 2).

Currently, there is no data as yet on the risk-benefit ratio of increased breast surveillance among patients with pathogenic variants in genes of moderate penetrance (e.g., *CHEK2*, *ATM* and *BLM*). There is remaining uncertainty in penetrance estimates for such variants, and, therefore, the optimal breast screening protocol and age of initiation remain unknown thus limiting the clinical utility of panel testing (for the present) to highly penetrant mutations. To better understand the role of these moderately penetrant genes will require population-based studies of mutation penetrance and clinical trials of risk-reducing interventions to guide clinical decisions. It is a major concern that while the practice of clinical cancer genetics is largely limited in developed countries to trained clinical cancer geneticists, this is not the case for the rest of the world.

The discovery of VUS that do not contribute to risk, may prompt anxiety and overtreatment particularly if the managing clinician is unfamiliar with genetics. Although our experience of finding ~3 VUS per gene is consistent with that from other studies,^8^ it also highlights the fact that the more we sequence, the more VUS we will unravel. This is particularly so in a population like Singapore, where we have multi-ethnic minority groups for whom there is limited publicly available sequencing data for variant reclassification. In the present study, consistent with our IRB–approved protocol, we did not re-contact any patient about VUS as there are no immediate clinical implications or recommendations to convey. In the clinical setting, where VUS results will be reported back to the patient, it is critical therefore that multigene panel testing is conducted in a dedicated genetics service with a genetics team familiar with cancer risk assessment and who are able to provide adequate pretest and post-test counselling.^9^

This study was conducted within a formal clinical cancer genetics practice adherent to evidence-based testing guidelines, and using the definition of pathogenic variants as recommended by the American College of Medical Genetics.^10^ With the clinical availability of multiple-gene sequencing panels and the concurrent decreasing cost of panel testing, it is anticipated that an increased demand for such gene-directed risk stratification will occur. These genetic testing costs are borne by the patient and not by any third-party payer, especially in Asian countries with no insurance coverage or government subsidies for genetic testing for most countries at present. With the reducing costs of genetic testing, many of these health policies are ripe for review if we wish to harness the power of gene-enabled care.

Our study has limitations. The 25 genes that we selected reflect published literature but an optimal multiple-gene panel for routine diagnostic use remains to be defined. Patients were enrolled from within a specialized clinical cancer genetics service and do not reflect general oncology practice nor the general population at large.

To the best of our knowledge, our study is the first to describe multiple-gene testing in an Asian setting within a formal clinical cancer genetics service. Although further research is required to guide practice, our study may help provide a framework for the clinical relevance of multiple-gene sequencing in cancer-risk assessment for other nascent centres in Asia embarking on multigene testing for patients referred for hereditary breast and ovarian cancer syndrome.

## Materials and Methods

### Patients

We studied 220 cases referred to the Cancer Genetics Service at the National Cancer Centre Singapore. Of these, 210 had a personal history of breast and/or ovarian cancer (192 had breast cancer, 9 had ovarian cancer, and 9 had breast and ovarian cancer). The subjects fulfilled at least one of the following criteria: (1) having a family history of breast and/or ovarian cancer in first- and/or second-degree relatives; (2) having breast and ovarian cancer in the same individual or bilateral breast cancer; (3) having early-onset breast cancer or ovarian cancer (⩽40 years of age). Clinical information including personal and family cancer histories, cancer histology and receptor status, were retrieved from case notes and clinical databases. All patients consented to participate in this study, which was approved by the SingHealth Centralized Institutional Review Board (CIRB 2008/435/B; CIRB 2010/406/B).

### Mutation detection using next-generation sequencing (NGS)

An optimised in-house method was used to extract DNA from peripheral blood.^5,11^ Capture was performed using the SureSelect XT2 target enrichment kit (Agilent, Santa Clara, CA, USA), targeting 25 genes (Supplementary Table 1). The Covaris S2 system (Covaris, Woburn, MA, USA) was used to fragment the genomic DNA samples as recommended by the manufacturer. The exome-enriched libraries were sequenced on the Illumina HiSeq platform (San Diego, CA, USA), with 100-bp paired-end reads.

### Deletion/duplication analysis

Detection of large genomic rearrangements in the *BRCA1* and *BRCA2* genes was done for all 220 samples using the Multiplex Ligation-dependent Probe Amplification test kits (P002-C2 BRCA1 and P045-BRCA2/CHEK2) and confirmation kits (P087-BRCA1 and P077-BRCA2; MRC-Holland, Amsterdam, Netherlands). DNA fragment analysis was performed on the ABI 3130 Genetic Analyzer (ABI-Life Technologies, Thermo Fisher Scientific Corporation, MA, USA) and analysed using the Coffalyser freeware v.131123.1303 (MRC-Holland).

### Bioinformatic analysis

The raw reads were aligned to the hg19 reference genome using BWA.^12^ BAM files were processed to identify variants using the Genome Analysis Tool kit. The variants were annotated using the ANNOVAR tool.^12^ The mean depth of coverage was ×315 (range: ×97–858). Population allele frequencies were extracted from the Exome Variant Server (http://evs.gs.washington.edu/EVS), 1000 Genomes (http://www.1000genomes.org), and dbSNP (http://www.ncbi.nlm.nih.gov/projects/SNP). Frameshift and nonsense mutations were considered to be deleterious. Missense variants were classified as damaging or benign using predictions from SIFT,^13^ PolyPhen-II HDIV,^14^ PolyPhen-II HVAR,^14^ LRT and Mutation Taster.^15^ If three or more of the five tools predicted the missense mutation to be damaging, then the mutation was classified as damaging. All deleterious or damaging variants were verified visually using the Integrative Genomics Viewer (IGV; Broad Institute), and collectively classified as pathogenic variants.

Variants that were synonymous, or classified as benign, unknown, uncertain or unspecified in the Breast Cancer Information Core, HGMD, ClinVar databases, were excluded. Also excluded were variants with an allele frequency greater than 1% as documented in the Exome Variant Server, 1000 Genomes, dbSNP and ExAC databases. All remaining variants were classified as VUS, and were verified visually using IGV.

### Validation of variants detected by NGS

All frameshift, nonsense and damaging missense mutations were validated by Sanger sequencing. PCR amplification using HotstarTaq (Qiagen, Hilden, Germany) using primers flanking mutations was performed as previously described.^11^ The BigDye Terminator v3.1 cycle sequencing kit (ABI-Life Technologies, Thermo Fisher Scientific Corporation) was used for the incorporation of dye-labelled dNTPs followed by Sanger sequencing using a 3130xl Genetic Analyzer (ABI-Life Technologies, Thermo Fisher Scientific Corporation). The chromatograms were visualised using the Seqman Pro v.12 (Lasergene; DNASTAR, Madison, WI, USA) software.

### Statistical analysis

Participant characteristics and sequencing results were tabulated, with descriptive statistics including medians, means and ranges.

## Figures and Tables

**Table 1:** Characteristics of the study participants

*Characteristics*	*Study participants (*n*=220)*	
	*No. of participants*	*%*
*Race/ethnicity*		
Chinese	181	82
Malay	17	8
Indonesians	7	3
Indians	5	2.5
Sri Lankan	1	0.5
Vietnamese	3	1
Burmese	1	0.5
Filipino	1	0.5
Japanese	1	0.5
Eurasian	1	0.5
Other races	2	1
		
*Personal history of breast cancer*		
Unilateral	177	80
Bilateral	18	8
		
*Age at first breast cancer diagnosis, years*		
Mean	39	
Median	37	
Range (Unknown age for 4 patients)	19–72	
		
Personal history of ovarian cancer	19	9
		
*Age at ovarian cancer diagnosis, years*		
Mean	46	
Median	50.5	
Range (unknown age for 3 patients)	15–65	
		
Family history of breast cancer	104	47
Family history of ovarian cancer	16	7

**Table 2:** Pathogenic variants with their Manchester and Boadicea scores

*ID*	*Race*	*Ca site*	*Subtype*	*Age at diagnosis (years)*	*Affected gene*	*Nucleotide change*	*Type of mutation*	*Amino-acid change*	*Family Ca history*	*MC score*	*Bo BRCA1*	*Bo BRCA2*	*Ref*
119	C	Bil Br Ca Histology Unk/Ov Ca	Unk	Br Ca (50)/Ov Ca (52)	*BRCA1*	c.3381T>A^a^	N	p.Y1127*^a^	Sis Br Ca (37)	46	64.6	22.1	
153	C	Ov Ca, Serous type		50	*BRCA1*	c.3381T>A^a^	N	p.Y1127*^a^	Sis Br Ca (40); Sis Ov Ca (60); Fa Thy Ca (54); Co Pat Br Ca (40)	46	33.2	10.2	6
121	C	Br IDC	ER−/PR−/Her2-	35	*BRCA1*	c.67_68delinsAG	Fr_ins	p.E23Rfs*18	Mo Br Ca (43); GM Mat Br Ca (45); Au Mat Br Ca (45); Au Mat Ov Ca (50)	75	22.6	4.2	6,16–19
152	V	Ov Ca, Endometrioid type		47	*BRCA1*	c.67_68delinsAG	Fr_ins	p.E23Rfs*18	Sis Ov Ca (47)	46	27.2	1	16–19
163	C	Bil Br IDC	ER−/PR−/Her2-	38, 46	*BRCA1*	c.3333delA	Fr_del	p.E1112Nfs*5	GM Mat Ov Ca (40)	42	32.2	6.1	20
166	C	No personal Ca history, Predictive testing	NA	NA	*BRCA1*	c.3333delA	Fr_del	p.E1112Nfs*5	Mo Bil Br Ca (38,46), Great GM Ov Ca (40)	Unk	Unk	Unk	20
12522596^b^	C	Br IDC	Unk	32	*BRCA1*	c.5072C>A	Mis	p.T1691K	Unk FH	Unk	Unk	Unk	21
FH83	C	Bil Br IDC/Atypical medullary type	ER−/PR−/Her2-	39, 46	*BRCA1*	c.5072C>A	Mis	p.T1691K	Twin Sis Bil Br Ca (30s, 40s); Sis Br Ca (40s)	51	88.8	9.7	21
104^b^	C	Br IDC	ER+/PR+/Her2-	33	*BRCA1*	c.5068A>C	Mis	p.K1690Q	Sis Thy Ca (31), Sis leukaemia (18)	1	0.5	2.1	5,22
YP61	C	Br IDC	ER−/PR−/Her2-	37	*BRCA1*	c.4327C>T	N	p.R1443*	Mo Br Ca (63), Au Mat Ov Ca (50)	30	10.1	3.3	6,23
172	M	Br IDC	ER−/PR−/Her2-	59	*BRCA1*	c.4065_4068del	Fr_del	p.N1355Kfs*10	Sis Br (49), Fa Col Ca (70), Au Mat Ga Ca	2	4.5	20.7	24
HR0039	C	Bil Br IDC	ER−/Pr−/Her2 Unk	41, 51	*BRCA1*	c.3858_3867del^a^	Fr_del	p.S1286Rfs*20^a^	Sis Br (49), Sis Br (43), Sis Br (30)	46	59.3	13.1	6
FH26	C	Br IDC	ER+/PR+/Her2-	57	*BRCA1*	c.3424delG^a^	Fr_del	p.A1142Hfs*13^a^	Sis Br Ca (43), Sis Br Ca (48), Mo Ga Ca (63)	14	2.8	5.7	6
137	IO	Br IDC	ER−/PR−/Her2-	39	*BRCA1*	c.3214delC	N	p.L1072fs*	Mo Ov Ca (46), Au Mat Br Ca (65), Au Mat Ov Ca (50)	63	50.4	1.6	25
159	I	Bil Br IDC	ER−/PR−/Her2+	22, 38	*BRCA1*	c.2766delA	Fr_del	p.V923Lfs*77	Au Mat Br Ca (45), Gr Mat Pa (60)	34	18.6	6.8	6,26
65	C	Br IDC (53), Serous Ov Ca (44), Pa Ca (51)	ER−/PR−/Her2-	44, 51, 53	*BRCA1*	c.2635G>T	N	p.E879*	No FH Ca	22	85.6	1	6,27
61	M	Br Ca Unk type	ER+/PR+/Her2 Unk	34	*BRCA1*	c.2145A>T	Mis	p.R762S	No FH Ca	1	2	1.8	
103	M	Bil Br IDC	ER+/PR+/Her2-	24, 30	*BRCA1*	c.981_982del	Fr_del	p.C328*	No FH Ca	22	18.7	33.2	28
150^b^	B	Br ILC	ER−/PR−/Her2-	40	*BRCA1*	c.745delA^a^	Fr_del	p.T249Lfs*49^a^	Au Pat Br Ca (50), Au Pat Br Ca (59)	10	4.4	0.9	6
59	C	Br mixed IDC ILC	ER+/PR+/Her2+	28	*BRCA1*	c.172C>G	Mis	p.P58A	Sis Ov Ca (40), Sis Ov Ca (46)	71	26.2	0.5	5
FH42	C	Br IDC	ER−/PR+/Her2-	43	*BRCA1*		Del	Deletion of Exons 16-19^a^	Mo Br Ca (30)	10	3	3.6	6
MR0017	C	Br IDC	ER−/PR−/Her2-	41	*BRCA1*		Dup	Duplication of Exon 13^a^	Unk FH	Unk	Unk	Unk	5,6
79	C	Br DCIS/Ov Ca	ER+/PR−/Her2-	38	*BRCA1*	c.442-15del10^a^	SE	Stop 182^a^	Sis Br and Ov Ca (55); Sis Br Ca(56)	38	68.1	1.7	5,6
MR0027	C	Br IDC	ER+/PR+/Her2-	36	*BRCA2*	c.483T>G	Mis	p.C161W	Au Pat Br Ca (40); Au Mat Br Ca (40)	10	3.3	0.7	5
FH87	C	Br Ca	ER+/PR+/Her2-	31	*BRCA2*	c.483T>G	Mis	p.C161W	Mo Br Ca (40); Co Mat Br Ca (40)	22	9.3	8.7	5
FH60	C	Br IDC	ER+/PR+/Her2-	56	*BRCA2*	c.2275delC^a^	Fr_del	p.L759Ffs*13^a^	Sis Br Ca (37), Fa Br Ca (72)	26	1.6	44.8	6
YP33	C	Br IDC	ER−/PR−/Her2-	40	*BRCA2*	c.3847_3848delGT	Fr_del	p.V1283Kfs*2	No FH Ca	1	4.3	1.1	6,29
168	C	No Ca	NA	NA	*BRCA2*	c.4151delT	Fr_del	p.1384Cfs*4	Mo Br Ca (42), Mo Ov Ca (50), Au Mat Ov Ca (40)	NA	NA	NA	5
HR0029	C	Br IDC	ER+/PR+/Her2-	51	*BRCA2*	c.5576_5579delTTAA	Fr_del	p.I1859Kfs*3	Sis Br Ca (53), Sis Br Ca (60), Sis Br Ca (51), Au Mat Br Ca (60)	18	1.4	2.2	6,30
151	C	Clear Cell Ov Ca		51	*BRCA2*	c.5799_5802delCCAA	Fr_del	p.N1933Kfs*29	Mo Br Ca (50), Au Mat Br Ca (60), Un Mat Ga Ca (50)	30	0	37	6,31
162	F	Br IDC	ER+/PR+/Her2-	36	*BRCA2*	c.6491delA^a^	Fr_del	p.Q2164Rfs*4^a^	No FH Ca	1	1.8	1.7	6
YP16^b^	C	Br IDC	ER+/PR+/Her2-	38	*BRCA2*	c.6986C>T	Mis	p.P2329L	No FH Ca	1	1.6	1.6	25
164	C	Br IDC, childhood acute leukaemia, meningiomas	ER+/PR+/Her2+	32	*BRCA2*	c.7480C>T	N	p.R2494*	No FH Ca	1	0.8	2.8	32
99	C	Br IDC	ER−/PR−/Her2-	42	*BRCA2*	c.7522G>A	Mis	p.G2508S	Mo Br Ca (80), Mo Col Ca (80), Au Mat Br Ca (70), Au Mat Ga Ca (70), Au Mat Br Ca (60)	2	0.4	0.5	33,34
HR0045^b^	M	Br IDC	ER+/PR−/Her2-	28	*BRCA2*	c.7631G>A	Mis	p.G2544D	Mo Br Ca (50), Au Mat Br Ca (60), Un Mat Ga Ca (50)	14	7.4	6.8	5
FH29	C	Br IDC	ER+/PR+/Her2-	49	*BRCA2*	c.7696_7697insA	Fr_ins	p.D2566Efs*5	Sis Br Ca (50)	2	0.6	2.1	6,35
LR0023	C	Br IDC	ER−/PR−/Her2-	36	*BRCA2*	c.8889_8891insA^a^	Fr_ins	p.A2964Kfs*54^a^	Co Br Ca (44)	10	1.8	1.7	6
FH53^b^	C	Br IDC	ER+/PR+/Her2-	41	*BRCA2*	c.8914delT^a^	Fr_del	p.L2972Cfs*4^a^	Mo Br Ca (50)	2	1	4.6	6
104^b^	C	Br IDC	ER+/PR+/Her2-	33	*BRCA2*	c.9294C>G	N	p.Y3098*	Sis Thy Ca (31), Sis leukaemia (18)	1	0.5	2.1	36
64	C	Br IDC/Ov Ca	ER Unk/PR Unk/Her2 Unk	18	*BRCA2*	c.7617+1G>A^a^	SE	Deletion of Exon 15^a^	HBOC (Br Ca, Ov Ca)	18	Unk	Unk	5,6
YP6^b^	C	Br IDC	ER+/PR+/Her2-	25	*PALB2*	c.113C>G	Mis	p.A38G	2 Others Ca non related	6	Unk	Unk	
YP59	C	Br IDC	ER+/PR+/Her2-	34	*PALB2*	c.113C>G	Mis	p.A38G	Un Pat Col Ca (40); Co Pat Ov (30)	30	Unk	Unk	
149	IO	Bil Serous Ov Carcinoma		59	*PALB2*	c.3166C>T^a^	N	p.Q1056*^a^	Sis Br Ca (61), Mo Br Ca (69)	46	Unk	Unk	4
LR0032	C	Br IDC	ER+/PR+/Her2+	24	*PALB2*	c.2607delC^a^	Fr_del	p.V870*^a^	No FH Ca	6	Unk	Unk	
120	C	Br Mixed IDC ILC	ER+/PR+/Her2-	39	*PALB2*	c.2411_2412delCT	Fr_del	p.S804Cfs*10	Sis Br Ca (35)	26	Unk	Unk	4
155^c^	I	Br IDC+mucinous Carcinoma	ER+/PR+/Her2-	54	*PALB2*	c.1448C>G^a^	N	p.S483*^a^	No FH Ca	14	Unk	Unk	4
LR0026^b^	C	Br IDC	ER+/PR+/Her2-	29	*PALB2*	c.1408delA^a^	Fr_del	p.T470Qfs*15^a^	No FH Ca	6	Unk	Unk	
YP19	C	Br IDC	ER−/PR−/Her2+	39	*PALB2*	c.3054G>C	Mis	p.E1018D	No FH Ca	1	Unk	Unk	
LR0009	C	Bil Br Ca, right chest wall myofibroblastic sarcoma, Pa Ca	ER−/PR−/Her2+	26	*TP53*	c.819delC^a^	Fr_del	p.S274Afs*38^a^	Bro sarcoma (38), Mo Ov Ca (38), Au Pat gastric Ca (68), GM Pat Ga Ca (72)	55	Unk	Unk	
131	C	Br IDC 32, Malignant Fibrous Histiocytoma of the subcutis (43), GIST of the stomach wall (43), several lumps	ER Unk/PR Unk/Her2 Unk	32	*TP53*	c.616G>A	Mis	p.G206S	Co Mat Br Ca (33)	14	Unk	Unk	37–39
158^b^	IO	Mixed invasive Br Ca	ER−/PR−/Her2+	30	*TP53*	c.356G>T^a^	Mis	p.R119L^a^	Mo Br Ca (49)	18	Unk	Unk	
HR0054	M	Br IDC	ER−/PR−/Her2-	32	*TP53*	c.331_343del^a^	Fr_del	p.T111Afs*16^a^	Mo Br Ca (34), Sis Brain tumour (10)	22	Unk	Unk	
158^b^	IO	Mixed invasive Br Ca	ER−/PR−/Her2+	30	*TP53*	c.275A>G^a^	Mis	p.N92S^a^	Mo Br Ca (49)	18	Unk	Unk	
980221	C	Br Ca	ER+/PR+/Her2 Unk	34	*TP53*	c.802+1G>A^a^	SE		Unk FH	Unk	Unk	Unk	
FH53^b^	C	Br IDC	ER+/PR+/Her2-	41	*CHEK2*	c.667C>T	Mis	p.R223C	Mo Br Ca (50)	2	Unk	Unk	
HR0045^b^	M	Br IDC	ER+/PR−/Her2-	28	*CHEK2*	c.667C>T	Mis	p.R223C	Mo Br Ca (50)	14	7.4	6.8	
LR0026^b^	C	Br IDC	ER+/PR+/Her2-	29	*ATM*	c.8800A>G	Mis	p.T2934A	No FH Ca	6	Unk	Unk	
YP62	C	Br IDC	ER+/PR+/Her2-	38	*PTEN*	c.641delA^a^	Fr_del	p.Q214Rfs*7^a^	Au Mat Br (30), Un Mat Pros (60)	22	Unk	Unk	
146	C	Multifocal Ov Ca, Br IDC, Endo Ca 50	ER+/PR+/Her2-	54	*PTEN*	c.672dup^a^	Fr_ins	p.Y225Ifs*18^a^	Fa Col Ca (60), Co Mat Col Ca (30)	1	Unk	Unk	
60	C	Unk type Br Ca, Neurofibromatosis	Unk	33	*NF1*	c.6480_6490del^a^	Fr_del	p.K2160Nfs*5^a^	No FH Ca	1	Unk	Unk	
150^b^	B	Br ILC	ER−/PR−/Her2-	40	*CDH1*	c.2359G>A^a^	Mis	p.V787I^a^	Au Pat Br Ca (50), Au Pat Br Ca (59)	10	4.4	0.9	
YP46	C	Br IDC	ER+/PR+/Her2-	33	*CDH1*	c.1888 C>G	Mis	p.L630V	GF Mat Ga Ca (70), GF Mat Pros Ca (70)	2	Unk	Unk	34
150^b^	B	Br ILC	ER−/PR−/Her2-	40	*CDKN2A*	c.221A>C	Mis	p.D74A	Au Pat Br Ca (50), Au Pat Br Ca (60)	10	4.4	0.9	
YP43	C	Br IDC	ER−/PR−/Her2-	31	*MLH1*	c.2135G>T	Mis	p.W712L	Au Mat Other Ca (53)	1	Unk	Unk	
YP6^b^	C	Br IDC	ER+/PR+/Her2-	25	*MLH1*	c.1153C>T	Mis	p.R385C	2 Other Ca Unk	6	Unk	Unk	40
167	C	Bil Br IDC	ER+/PR+/Her2-	52	*MSH6*	c.2362A>G^a^	Mis	p.I788V^a^	Fa Col Ca (70)	1	Unk	Unk	
YP28	C	Br IDC	ER+/PR+/Her2-	39	*MSH6*	c.2891G>A^a^	Mis	p.C964Y^a^	Unk FH	Unk	Unk	Unk	
170	SL	Br IDC	ER−/PR−/Her2-	38	*MSH6*	c.3227G>A	Mis	p.R1076H	Mo Br Ca (39)	14	12.7	3.1	
142	J	No Ca	NA	NA	*PMS2*	c.944G>T^a^	Mis	p.R315L^a^	Au Mat Br Ca (48), GM Mat Br Ca (60)	Unk	Unk	Unk	
86	C	Unk type Br Ca	ER+/PR+/Her2+	30	*BARD1*	c.1298A>G	Mis	p.H433R	Mo Br Ca (45)	10	Unk	Unk	
YP44	C	Br IDC	ER−/PR+/Her2-	37	*BRIP1*	c.1442G>A	Mis	p.G481D	Au Mat Br Ca (40)	10	Unk	Unk	
990493^b^	C	Br IDC with mucinous differentiation	ER+/PR−/Her2 Unk	35	*BRIP1*	c.2440 C>T	Mis	p.R814C	Unk FH	Unk	Unk	Unk	
990493^b^	C	Br IDC with mucinous differentiation	ER+/PR−/Her2 Unk	35	*RAD51C*	c.635G>A	Mis	p.R212H	Unk FH	Unk	Unk	Unk	34
YP5	C	Br IDC	ER+/PR+/Her2-	38	*RAD51D*	c.932T>A	Mis	p.I311N	FH not found in the Case note	Unk	Unk	Unk	
YP16^b^	C	Br IDC	ER+/PR+/Her2-	38	*RAD51D*	c.932T>A	Mis	p.I311N	No FH Ca	1	Unk	Unk	41
YP47	C	Br IDC	ER−/PR−/Her2+	36	*RAD51D*	c.932T>A	Mis	p.I311N	FH not found in the Case note	Unk	Unk	Unk	41
12522596^b^	C	Br IDC	Unk	32	*RAD51D*	c.932T>A	Mis	p.I311N	Unk FH	Unk	Unk	Unk	41

Abbreviations: Au, aunt; B, burmese; Bil, bilateral; Bo, boadicea Score; Br, breast; Bro, brother; C, chinese; Ca, cancer; Co, cousin; Col, colorectal; Endo, endometrial; ER, oestrogen receptor; F, filipino; Fa, father; FH, family history; Fr_del, frameshift deletion; Fr_ins, frameshift Insertion; ga, gastric; GF, grandfather; GM, grandmother; GIST, gastrointestinal stromal tumour; I, Indian; IDC, invasive ductal carcinoma; ILC, invasive lobular carcinoma; IO, indonesian; J, japanese; M, malay; Mis, missense; Mat, maternal; MC, manchester Score; Mo, mother; N, nonsense; NA, not applicable; Ov, ovarian; Pa, pancreatic; Pat, paternal; Pros, prostate; PR, progesterone receptor; Ref, reference; SE, splice site Error; Sis, sister; SL, sri lankan; Thy, thyroid; Un, uncle; Unk, unknown; V, vietnamese.

a Underlined indicates novel pathogenic variants identified by our group.

b Patients with more than one pathogenic variant.

c Patient with male breast cancer.

**Table 3:** Mean, median and range of Manchester Scores in cases grouped according to *BRCA1* and *BRCA2* mutation status

	*BRCA1*	*BRCA2*	*Either BRCA1 or BRCA2*	*Mutation positive for other genes*	*No mutations*
Mean	34	10	23	13	9
Median	36	2	18	9	2
Range	1–75	1–30	1–75	1–55	1–71
