# Supplementary material for: Inherited breast cancer predisposition in Asians: multigene panel testing outcomes from Singapore
Source: NPJ Genom Med. 2016 Jan 13;1:15003–. doi: 10.1038/npjgenmed.2015.3 (PMC5685290; doi:10.1038/npjgenmed.2015.3)
Supplement: Supplementary Table 1 [file npjgenmed20153-s1.doc]

Supplementary table 1. Description of the 25 targeted genes in this study

|  | **Gene** | **mRNA Transcript ID** |
| --- | --- | --- |
| 1 | ATM | NM_000051.3 |
| 2 | BARD1 | NM_000465.3 |
| 3 | BMPR1A | NM_004329.2 |
| 4 | BRCA1 | NM_007294.3 |
| 5 | BRCA2 | NM_000059.3 |
| 6 | BRIP1 | NM_032043.2 |
| 7 | CDH1 | NM_004360.3 |
| 8 | CDKN2A | NM_001195132.1 |
| 9 | CHEK2 | NM_001005735.1 |
| 10 | FANCC | NM_000136.2;  NM_001243744.1(R484Q only) |
| 11 | MLH1 | NM_000249.3 |
| 12 | MSH2 | NM_000251.2 |
| 13 | MSH6 | NM_000179.2 |
| 14 | NBN | NM_002485.4  no deleterious/VUS detected |
| 15 | NF1 | NM_001042492.2 |
| 16 | PALB2 | NM_024675.3 |
| 17 | PMS2 | NM_000535.5 |
| 18 | PTEN | NM_000314.4 |
| 19 | RAD51C | NM_058216.2 |
| 20 | RAD51D | NM_002878.3 |
| 21 | SMAD4 | NM_005359.5 |
| 22 | STK11 | NM_000455.4 |
| 23 | TP53 | NM_001276696.1 |
| 24 | VHL | NM_000551.3 |
| 25 | XRCC2 | NM_005431.1 |
